# Difference in suicide methods used between suicide attempters and suicide completers

**DOI:** 10.1186/1752-4458-8-54

**Published:** 2014-12-15

**Authors:** Meerae Lim, Sang Uk Lee, Jong-Ik Park

**Affiliations:** St. Andrea Neuropsychiatric Hospital, Icheon, Republic of Korea; Korea Suicide Prevention Center, Seoul, Republic of Korea; Department of Psychiatry, Kangwon National University School of Medicine, 17-1 Hyoja-3-Dong, Chuncheon-Si, Kangwon-Do 200-947 Korea

**Keywords:** Suicide, Suicide method, Lethality, Suicide attempters, Suicide completers

## Abstract

**Background:**

In light of the increased suicide rate in Korea, it has become important for researchers to examine the various factors associated with it. The purpose of this study is to compare and analyze the difference between suicide attempters and completers in terms of the suicide methods used and the lethality of these methods. In addition, we investigated certain demographic factors that are associated with the choice of suicide method by evaluating their lethality.

**Finding:**

The most frequently used methods of suicide were different in the two groups of attempters and completers. Drug poisoning was the most frequent method in suicide attempters, whereas hanging was the most common method among suicide completers. Drug poisoning, stabbing, and other chemical poisoning were evaluated as relatively non-lethal compared to other suicide methods. While about 70.0% of the suicide attempters used relatively non-lethal methods, almost all suicide completers used lethal methods, based on our classification of the lethality of the method. In terms of gender, males used more lethal methods of suicide.

**Conclusions:**

Suicide completers’ choice of suicide methods are different from those of suicide attempters and tend to be more lethal. Interventions to restrict access to more lethal suicidal methods could be a useful strategy to reduce the suicide rates in South Korea.

Suicide is a serious social problem in Korea. Data from Korea’s National Statistics Office published in September 2013 states that the total number of deaths in the previous year was 14,160 and that suicide was the fourth leading cause of death. Korea has the highest suicide rate i.e., 29.1 per 100,000, from the member countries of the Organization for Economic Cooperation and Development where the average suicide rate is 12.5 per 100,000 [1].

Little is known about why the suicide rate has increased so rapidly in Korea. The risk factors are very complex and interwoven. Therefore, we chose to focus on the lethality of the suicide methods used and their association with the increasing suicide rate. Previous research has pointed to the link between suicide methods and the rate of suicide. Spicer and Miller [2] showed that the particular suicide method chosen is strongly linked to suicide completion. Another study showed that the use of specific suicide methods such as hanging, is reflected in the increased suicide rate in Korea [3]. Studies on suicide in Asia suggest that the availability of fatal suicide methods can increase suicide rates [4].

The purpose of this paper is to compare and analyze the difference between suicide attempters and completers in terms of the suicide methods used. In addition, we investigated the association of certain demographic factors with the choice of suicide method in terms of their lethality.

In this study, we collected and analyzed two kinds of secondary data. First, we obtained the results of a nationwide survey on suicide attempters, which was done as a pilot study of Korean Nationwide Suicide Survey (KNSS) based on law. Data on suicide attempts that occurred nationwide between July 6, 2012 and November 25, 2012 were collected. A suicide attempt was defined as “a self-destructive behavior with intent to end one’s life independent of resulting damage” [5]. Attempters from this list who died upon arrival at the hospital were excluded from the analysis. Data were collected by reviewing the medical records in seven university hospitals: Boramae Medical Center, Seoul; Seoul National University Bundang Hospital, Gyeonggi-do; Kangwon National University Hospital, Gangwon-do; Eulgi University Hospital, Choonchung-do; Chonnam National University Hospital, Chonra-do; Busan National University Hospital, Gyeongsang-do; and Gueongsang National University Hospital, after obtaining ethical approval from the institutional review board (IRB).

The second data source was the “Statistics of cause of death, 2012” records which was public data published by Statistics Korea. We analyzed the data concerning deaths by suicide that occurred between July 6, 2012 and November 25, 2012, the same period under analysis in the previous data set of medical records of suicide attempts.

From these two data sets, we gathered information about age, gender, marital status, degree of education, and method of suicide or suicide attempt. In case of attempters, we could gather information about medical results of suicide attempt in addition to suicide methods. Medical results were evaluated by Colombia Suicide Severity Rating Scale (C-SSRS) lethality subscale [6].

Data were analyzed using SPSS 18.0 for Windows (SPSS Inc., Chicago, IL, USA). Each method of suicide was analyzed separately in relation to suicide attempt and suicide completion. We evaluated the lethality of each suicide method in two ways. First, we used the C-SSRS lethality subscale to rate lethality of each suicide method. With this scale, lethality is appraised on a six-point Likert scale, with responses ranging from 0 (*no physical damage*) to 5 (*death*) [6]. Second, we developed a “lethality ratio” that is defined as the ratio of the percentage of the specific method used by suicide completers to the percentage of the specific method used by suicide attempters. To examine the gender difference in the choice of suicide method, we used a binary logistic regression and adjusted for age, marital status, and level of education.

A total of 502 medical records of suicide attempters were included in this study. The average age of the participants was 43.05 years (±18.52), with ages ranging from 13 to 95 years. There were more females (58.4%, *n* = 293) than males (41.6%, *n* = 209).

Table 1 shows the frequency of each method of suicide for suicide attempters. The most commonly used method for suicide attempters was poisoning by prescribed drug overdose (49.6%, *n* = 248), followed by wrist cutting (15.4%, *n* = 77), and pesticide poisoning (14.8%, *n* = 74). Table 2 shows the gender differences in the choice of method for suicide attempts. While drug poisoning was the method preferred mostly in females, pesticide, herbicide, and gas poisoning were methods preferred in males.

**Table 1:** **Comparison of methods of suicide between suicide attempters and suicide completers**

	Suicide attempters		Suicide completers		Statistics
	N	%	N	%	
Drug poisoning	248	49.6	56	1.0	*χ* ^2^ = 2784.2^a^ *p* < .001
Gas poisoning	34	6.8	392	7.3	
Pesticide or herbicide	74	14.8	741	13.8	
Other chemical poisoning	20	4.0	138	2.6	
Hanging	25	5.0	2810	52.2	
Drowning	4	0.8	207	3.8	
Gun suicide	0	0.0	6	0.1	
Immolation	0	0.0	19	0.3	
Stabbing	77	15.4	35	0.6	
Jumping	18	3.6	953	17.7	
Others	0	0.0	32	0.6	
Total	500	100.0	5388	100.0	

^a^Drowning, gun suicide, immolation, others was excluded from the analysis because that expected frequencies were below than 5.

**Table 2:** **Comparison of methods of suicide by gender**

	Male		Female		Total	
	N	%	N	%	N	%
Drug poisoning	68	32.5	180	61.9	248	49.6
Gas poisoning	27	12.9	7	2.4	34	6.8
Pesticide or herbicide	52	24.9	22	7.6	74	14.8
Other chemical poisoning	7	3.3	13	4.5	20	4.0
Hanging	13	6.2	12	4.1	25	5.0
Drowning	2	1.0	2	0.7	4	0.8
Stabbing	33	15.8	44	15.1	77	15.4
Jumping	7	3.3	11	3.8	18	3.6
Total	209	100.0	291	100.0	500	100.0

Data from a total of 5,388 suicide completers were analyzed. The mean age of suicide completers was 52.36 years (±18.31), with ages ranging from 12 to 98 years. Unlike suicide attempters, there were more male (67.2%, N = 3621) than female (32.8%, N = 1767) suicide completers. There was a significant gender difference found between the suicide attempter and suicide completer groups (*χ*^2^ = 132.0, *df* = 1, *p* < .01).

Table 1 also shows the frequency of each method of suicide for suicide completers. The most commonly used method by suicide completers was hanging (52.2%, N = 2810), followed by jumping from a height (17.7%, N = 953), and pesticide poisoning (13.8%, N = 741). However, the percentage of methods such as drug poisoning and stabbing, which were the first and second most commonly used methods in suicide attempters, was low (1.0%, N = 56 and 0.6%, N = 35 respectively) in suicide completers.

As mentioned in the methods section, we evaluated lethality of each method of suicide in different two ways. According to the C-SSRS lethality subscale ratings, jumping from a height led to the most severe medical damage, followed by pesticide or herbicide poisoning. According to the lethality ratio, hanging was found to be the most lethal suicide method, followed by jumping from a height, and drowning. Drug poisoning and stabbing were relatively less lethal based on these two methods of evaluation (Table 3).

**Table 3:** **Lethality of the method used for attempting suicide**

	C-SSRS ^a^ Lethality subscale		Lethality ratio ^b^
	Mean	Standard deviation	
Drug poisoning	2.48	0.92	2.0
Gas poisoning	3.00	1.00	107.3
Pesticide or herbicide	3.44	1.16	93.2
Other chemical poisoning	2.00	0.85	65.0
Hanging	3.00	1.16	1044.0
Drowning^c^			475.0
Stabbing	2.39	0.98	3.9
Jumping	4.07	1.77	491.7

^a^C-SSRS: Colombia Suicide Severity Rating Scale.

^b^Lethality ratio: (% of the specific suicide method used by suicide completers)/(% of the specific suicide method used by suicide attempters).

^c^Among the 502 suicide attempters, 4 attempters used drowning as a suicide method. Only 1 participant from among them answered the C-SSRS lethality subscale. That particular score is not representative and therefore, we excluded this score from this table.

For further analysis, we divided the suicide methods used into two groups: lethal and non-lethal. Methods such as drug poisoning, stabbing, and other chemical poisoning, which accounted for relatively less lethal ratings in both evaluations were classified as non-lethal. The rest of the methods were classified as lethal. The analysis of these two groups based on lethality of method showed that 69.6% of the suicide attempters used non-lethal suicide methods, whereas 95.9% of the suicide completers used lethal methods (*χ*^2^ = 2089.5, *df* = 1, *p* < .01).

A binary logistic regression analysis was used to confirm the relationship between gender and choice of suicide method, after adjusting for age, marital status, and degree of education (Table 4). Results showed that males were significantly associated with using lethal suicide methods.

**Table 4:** **Comparison of the choice of suicide methods between males and females**

	Non-lethal method	Lethal method	Total	Exp(B)	95% C.I.
Male	101 (48.8%)	106 (51.2%)	207 (100.0%)	3.75	2.30 ~ 6.12
Female	224 (77.5%)	65 (22.5%)	289 (100.0%)	Reference	
Total	325 (65.5%)	171 (34.5%)	496 (100.0%)		

Co-variates: age, marital status, degree of education.

The present study is the first to compare the difference in the methods of suicide between suicide attempters and suicide completers in South Korea. The most frequently used methods of suicide were different for suicide attempters and completers. Drug poisoning was the most frequent used method in suicide attempters, whereas hanging was the most frequently used method in suicide completers. When we arbitrarily classified each method of suicide into one of two groups i.e., lethal and non-lethal, we found that around 70.0% of the suicide attempters used non-lethal methods and almost all suicide completers used lethal methods, based on our classification of lethality. This result is supported by previous research that showed that the choice of lethal suicide methods is associated with suicidal completion [2–4, 7, 8].

Another important result of our study is the gender difference between suicide attempters and completers. There were more males in the suicide completers group and more females in the suicide attempters group. This finding is consistent with some previous studies [9–13]. Our findings demonstrate that the disparity between suicide rates and the number of suicide attempts among males and females may be explained by their choice of suicide method.

According to Gunnell *et al*. [7], the methods of suicide commonly employed are influenced by their availability and access. They also reported that the accessibility and lethality of particular methods of suicide might have profound effects on overall suicide rates. In our study, we also emphasized that the rapid increase in suicide rates in Korea should be attributed in part to the change in suicide methods used over the past 15 years (Figure 1). In particular, the use of lethal suicide methods such as hanging and gas poisoning has increased recently. The rate of use of the suicide method of hanging has steadily increased from 31.4% in 2001 to 50.5% in 2012. In addition, the use of gas poisoning has increased 20 times in 2012 as compared to 2001 (from 0.4% in 2001to 8.5% in 2012). We still do not know the exact reasons for this increase and decrease in the usage of these suicide methods. However, we can hypothesize that the media has a role to play, especially the vivid and alluring descriptions of celebrity suicides may lead to the Werther effect thereby increasing the rate of copycat suicides using methods such as hanging and gas poisoning [14, 15].

**Figure 1:**
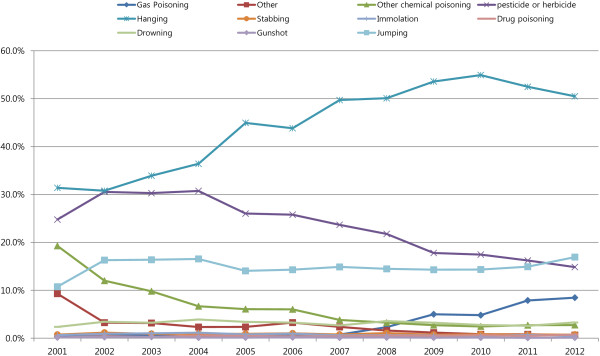
**Changes in the methods of suicide used in Korea across time.**
